# 

**Published:** 1978

**Authors:** 


JANUARY/APRIL 1978

VOLUME 93 (i/ii)

NUMBER 345/346

Published Quarterly

Annual Subscription £6 Post Free

### BRISTOL MEDICO-CHIRURGICAL JOURNAL

### Contents

3 Obituary—
Robert V. Cooke, M.D., Ch.M., F.R.C.S.

5 Richard Bright's Reports of Medical Cases (1827): A sesquicentennial note
Jeffrey Boss

7 Medical Care—more or less?
H. G. Mather, M.A., M.D., F.R.C.P.

Established 1883

### BRISTOL MEDICO-CHIRURGICAL JOURNAL

Incorporating the Medical Journal of the South West

### Editorial Committee

EDITOR
*Dr. D. S. Reeves*
*Southmead Hospital, Bristol, BS10 5NB*
ASSISTANT EDITOR
*Dr. A. P. Radford*
MEMBERS
*Dr. Rhys Davies*
*Dr. M. B. Lennard*
*Dr. D. W. Wright*
*The Hon. Treasurer of the Society*
*The Hon. Secretary of the Society*
SECRETARY
*Mr. B. P. Jones, The Library, Medical School,*
*University Walk, Bristol, BS8 1TD*

### Bristol Medico-Chirurgical Society

PRESIDENT 1977/78
*Dr. A. T. M. Roberts*
PRESIDENT ELECT
*H. K. Lucas, Esq.*
HONORARY SECRETARY
*A. J. Webb, Esq., 7 Percival Road, Bristol, BS8 3LE*
HONORARY TREASURER
*Dr. J. Penry, Southmead Hospital, Bristol, BS10 5NB*

The Society was founded in 1874. In 1883 publication of the Journal began and has continued quarterly ever since then. During the years 1949 to 1962 it appeared under the title of 'The Medical Journal of the South West'.

### Notice to Contributors

*The Journal is especially concerned to provide a place for the recording of medical thought, interests, and practice in and around Bristol. It therefore welcomes articles that, as well as being of immediate interest to members, will document the local and contemporary medical scene for our successors.*

*Original articles are invited provided that they have not been published elsewhere. Illustrations should be supplied ready for reproduction. Line drawings should be in Indian ink on firm white paper or board. The author will be informed if it is found necessary to ask him to pay for the printing of photographs etc.*

*The following contributions will be especially welcome; notes of forthcoming events, meetings held, degrees conferred, staff appointments, honours and public appointments and other local news.*

*Advice on preparation can be obtained from the Editor.*

Printed by the University of Bristol Printing Unit

### University of Bristol

### FACULTY OF MEDICINE

**The Degree of Master of Science in Medical Sciences**
**October 1977**

Louis ANASTASIADIS
Tun YI

**The Degree of Doctor of Philosophy**
**October 1977**

Christopher Miles BROWN
Patricia Ann TRUMPER
Avril Eira WATERMAN

**The Degree of Doctor of Medicine**
**October 1977**

John Brian Frederick GRANT
David Patrick McINERNEY

**The Degree of Doctor of Philosophy**
**January 1978**

Derek George BAGGOTT
Wilfred John LOCKYER
Vassiliky D. PETROCHEILOU
Sarah PRICHARD-THOMAS
Charles William TODD
Michael Ralph WILLS

**The Degree of Doctor of Medicine**
**January 1978**

Paul Nelson DURRINGTON
Edward John WAKLEY

**March 1978**

David Wallace BULLIMORE

**Final Examination for the Degree of B.D.S. (Section III)**
**November/December 1977**

**WITH HONOURS**

Maxine ARMSTRONG
Annabel Mary CLARKE
Maria Ann COCHRANE
Joan Judith ERRINGTON
David Alan WESTLEY

**PASS**

Elizabeth Louise BAILEY
Paul Michael BAINBRIDGE
Peter Martin BATEMAN
Martyn BEAN
Jeremy Peter BLOOMER
Helen Christine BOTT
Gillian Lesley BUTTERWORTH
Gillian Mary COLLARD
Geoffrey Dennis CURNOCK
Mark Jonathan DAVIES
Frances Jean DOWLER
Stephen Albert GOODA
Peter Alexander GORDON
Rodney Charles KENT
Jacqueline LARNER
Alasdair Gordon MILLER
Joseph Jolyon Dougil NEAL
Brian Robert OSBORNE
Robert William PAICE
Dennis Ian PEARCE
Stephen Michael PEARCE
Stephen Charles PRESTON
Roger Nii Yalai QUAYE
John Beverley SMALLEY
Antony Rupert VALLANCE
Alexandra Anne WILLIAMS

### A SELECTION OF RECENT ADDITIONS TO THE MEDICAL LIBRARY

ADAMS, D. H. & BELL, T. M.: Slow viruses. (Addison-Wesley)
ANDREWS, J. M. et al.: Amyotrophic lateral sclerosis: recent research trends. (Acad. Pr.)
BAILEY, H.: Emergency surgery. Ed.10. (Wright) Presented by John Wright & Sons Ltd.
BAJAJ, J. S.: Insulin and metabolism. (Excerpta Medica)
BASTRON, R. D. & DEUTSCH, S.: Anesthesia and the kidney. (Grune & Stratton)
BENCHIMOL, A.: Non-invasive diagnostic techniques in cardiology. (Williams & Wilkins)
BERKOVITZ, B. K. B. & MOXHAM, B. J.: Multiple choice questions in the anatomical sciences for students of dentistry. (Wright) Presented by John Wright & Sons Ltd.
BERLIN, N. I. et al.: Polycythemia. (Grune & Stratton)
BERTELLI, A. ed.: New trends in the therapy of liver diseases. (Karger)
BLOCK, M. H.: Text-atlas of hematology. (Lee & Febiger)
BOONE, D. C.: Comprehensive management of hemophilia. (F. A. Davis)
BRAAKMANN, R. & PENNINGS, L.: Injuries of the cervical spine. (Excerpta Medica)
BRANTHWAITE, M. A.: Anaesthesia for cardiac surgery and allied procedures. (Blackwell)
BRATTSTRÖM, M.: Principles of joint protection in chronic rheumatic disease. (Wolfe)
BROOKE, B. N. et al.: Crohn's disease. (Macmillan)
CHIRIGOS, M. A.: Control of neoplasia by modulation of the immune system. (Raven Pr.)
CLIFFORD, M. A. & DRUMMOND, A. E.: Radiographic techniques related to pathology. Ed.2. (Wright) Presented by John Wright & Sons Ltd.
DeGROOT, L. J. & STANBURY, J. B.: The thyroid and its diseases. Ed.4. (Mc.Graw-Hill)
DE FRANCE, J. F.: The septal nuclei. (Plenum Pr.)
DEVAS, M.: Geriatric orthopaedics. (Acad. Pr.)
DUNCAN, C. J.: Calcium in biological systems. (Cambridge Univ. Pr.)
FLACH, F. F. & DRAGHI, S. C.: The nature and treatment of depression. (Wiley)
FOWLER, N. O.: Cardiac arrhythmias: diagnosis and treatment. Ed.2. (Harper & Row)
FREUDENTHAL, R. I. & JONES, P.: Polynuclear aromatic hydrocarbons: chemistry, metabolism and carcinogenesis. (Carcinogensis, 1) (Raven Pr.)
FRIEDE, R. L.: Developmental neuropathology. (Springer)
FRIEDEWALD, V. E.: Textbook of echocardiography. (Saunders)
GABE, M.: Histological techniques. (Springer)
GARDNER, A. W. & ROYLANCE, P. J.: New advanced first-aid. Ed.2. (Wright) Presented by John Wright & Sons Ltd.
GOMPEL, C. & SILVERBERG, S. G.: Pathology in gynaecology and obstetrics. Ed.2. (Blackwell)
GORLIN, R.: Coronary artery disease. (Saunders)
GRUNDMANN, E. & VAHLENSIECK, W.: Tumors of the male genital system (Recent results in cancer research, 60). (Springer)
HICKS, D.: Primary health care: a review. (HMSO)
ILLIS, L. S.: Viral diseases of the central nervous system. (Baillière)
INGRAM, W. R.: A review of anatomical neurology. (Univ.Park Pr.)
ISHIKAWA, T. et al.: Growth and differentiation of micro-organisms. (Univ.Park Pr.)
JENNETT, B. et al.: Effect of carotid artery surgery on cerebral blood flow: clinical and experimental studies. (Excerpta Medica)
KATZ, A. M.: Physiology and biophysics of the heart. (Raven Pr.)
KELALIS, P. P. et al.: Clinical pediatric urology. 2 vols. (Saunders)
KOLBER, A. R. & KOHIYAMA, M.: Mechanism and regulation of DNA replication. (Plenum Pr.)
KRUGMAN, S. et al.: Infectious diseases of children. Ed.6. (Mosby)
LEWIN, S.: Vitamin C. (Acad. Pr.)
MACLEAN, D. & EMSLIE-SMITH, D.: Accidental hypothermia in man. (Blackwell)
MURPHY, P.: The neutrophil. (Plenum Pr.)
NELSON, J. H.: Atlas of radical pelvic surgery. Ed.2. (Appleton-Century-Crofts)
NIERLICH, D. P. et al. eds.: Molecular mechanisms in the control of gene expression. (Acad. Pr.)
OOSTERVELD, W. J.: Audio-vestibular system and facial nerve (Advances in oto-rhino-laryngology, 22). (Karger)
PERRY, S. V. et al.: Contractile system in non-muscle tissues. (North-Holland)
PYE, W.: Surgical handicraft. Ed.20, by J. Kyle. (Wright) Presented by John Wright & Sons Ltd.
RAMACHANDRAN, G. N. & REDDI, A. H.: Biochemistry of collagen. (Plenum Pr.)
RICCARDI, V. M.: The genetic approach to human disease. (Oxford Univ.Pr.)
ROSENBERG, A. & SCHENGRUND, C. L.: Biological roles of sialic acid. (Plenum Pr.)
ROSENTHAL, J. & FRANZ, H. E.: Medical and surgical aspects of renovascular hypertension. (Karger)
SCHOENBERG, B. S.: Multiple primary malignant neoplasms (Recent results in cancer research, 58). (Springer)
SIEGENTHALER, W. et al.: Diuretics in research and clinics. (Thieme)
SPILLER, G. A. & AMEN, R. J.: Fiber in human nutrition. (Plenum Pr.)
SPIRO, H. M.: Clinical gastroenterology. Ed.2. (Baillière)
TAYLOR, C. R.: Hodgkin's disease and the lymphomas. (Eden Pr.)
TEILUM, G.: Special tumors of ovary and testis. Ed.2. (Munksgaard)
TER MEULEN, V. & KATZ, M.: Slow virus infections of the CNS. (Springer)
THOMPSON, R. A. & GREEN, J. R.: Infectious diseases of the central nervous system. (Raven Pr.)
THOMSON, W. A. R.: A dictionary of medical ethics and practice. (Wright) Presented by John Wright & Sons Ltd.
USDIN, E. et al.: Neuroregulators and psychiatric disorders. (Oxford Univ.Pr.)
VOLLMAN, R.: The menstrual cycle. (Saunders)
WRIGHT, V. & MOLL, J. M. H.: Seronegative polyarthritis. (North-Holland)

